# A Novel F5 Gene Variant Associated With Early-Life Ischemic Brain Injury in a Child With a Familial Coagulation Disorder

**DOI:** 10.7759/cureus.108044

**Published:** 2026-04-30

**Authors:** Sara S Hassanien, Ehab Hanafy, Badriah G Alasmari, Mohamed Mansour, Ayman Abualama

**Affiliations:** 1 Oncology Center, Armed Forces Hospital Southern Region, Khamis Mushayt, SAU; 2 Pediatrics, Armed Forces Hospital Southern Region, Khamis Mushayt, SAU

**Keywords:** activated protein c resistance, arterial ischemic stroke, f5 gene variant, inherited thrombophilia, ischemic brain injury, pediatric hemiparesis, sickle cell trait, whole exome sequencing

## Abstract

Perinatal and early-life arterial ischemic stroke are important causes of hemiparesis and lifelong neurodevelopmental morbidity. While many cases are considered idiopathic, inherited thrombophilia is increasingly implicated in selected patients.

We report an infant who developed right-sided motor impairment recognized at nine months of age. He was born at term after an uncomplicated pregnancy, although the neonatal period was complicated by transient respiratory distress and a small atrial septal defect requiring brief neonatal intensive care admission. Examination demonstrated right spastic hemiparesis, delayed speech development, mild dysmorphic facial features, and bilateral fifth-finger clinodactyly. Brain magnetic resonance imaging showed left-sided cerebral and cerebellar volume loss with a focal left thalamic lesion, consistent with a remote ischemic insult. Hematologic evaluation identified sickle cell trait. Whole-exome sequencing revealed a heterozygous c.3482G>A (p.Arg1161Gln) variant in the F5 gene, associated with activated protein C resistance and classified as a variant of uncertain significance. No recurrent thrombotic events have occurred to date, and the patient remains clinically stable under conservative hematologic follow-up.

This case supports the concept that early-life ischemic brain injury may arise from the interaction of multiple prothrombotic factors rather than a single isolated abnormality. The coexistence of sickle cell trait and a rare F5 variant associated with activated protein C resistance may have contributed to a lowered thrombotic threshold in this patient. Comprehensive thrombophilia and genetic evaluation may therefore provide important diagnostic and risk-stratification value in children with otherwise unexplained early ischemic stroke.

## Introduction

Pediatric arterial ischemic stroke (AIS) and perinatal cerebrovascular injury are increasingly recognized as important causes of long-term neurological morbidity, accounting for significant rates of hemiparesis, developmental delay, epilepsy, and cognitive impairment in childhood [1]. Unlike adult stroke, the etiopathogenesis of pediatric cerebrovascular events is frequently multifactorial, involving a complex interplay between perinatal factors, structural cardiac abnormalities, systemic illness, and genetic predisposition.

Despite advances in neuroimaging and neonatal care, a substantial proportion of pediatric ischemic brain injuries remain unexplained, particularly in children without clear perinatal complications or acquired risk factors [2]. In this context, inherited thrombophilia has gained attention as a potential contributor to early-life cerebrovascular events. Genetic variants affecting the coagulation cascade may predispose susceptible individuals to cerebral ischemia during critical developmental periods.

Among inherited prothrombotic conditions, variants in the F5 gene, most notably those associated with activated protein C resistance, represent some of the most commonly identified genetic risk factors for thrombosis in both pediatric and adult populations [3]. Activated protein C resistance refers to reduced anticoagulant response to activated protein C, resulting in increased thrombin generation and a greater tendency toward thrombosis. Although the pathogenic significance of heterozygous F5 variants in childhood stroke remains variable and incompletely understood, accumulating evidence suggests that their presence may increase cerebrovascular vulnerability, particularly when additional risk modifiers coexist.

Sickle cell trait is generally considered a benign carrier state; however, emerging data indicate that it may confer a modest but measurable prothrombotic and vascular risk under certain physiological or environmental conditions [4]. The coexistence of sickle cell trait with other inherited thrombophilic factors may therefore represent an underrecognized risk constellation in pediatric cerebrovascular disease.

In this report, we describe a child with early-onset hemiparesis and neuroimaging findings consistent with a remote ischemic brain insult, in whom genetic evaluation revealed a heterozygous F5 gene variant associated with activated protein C resistance, alongside sickle cell trait. This case highlights the diagnostic value of comprehensive etiological evaluation, including genetic testing, in unexplained pediatric cerebrovascular injury and underscores the importance of individualized thrombosis risk assessment and long-term follow-up.

## Case presentation

A one-year-old boy, the second child of non-consanguineous parents, was referred for evaluation of longstanding right-sided motor weakness. Family history was notable for one female sibling with factor V deficiency and a bleeding tendency, while another male sibling was healthy with no known hematologic disorder. The patient’s father was a known carrier of sickle cell trait.

The patient was clinically stable at presentation, alert, and active. Physical examination revealed mild dysmorphic facial features, including a wide nasal bridge and epicanthic folds. Neurological examination demonstrated right-sided spastic hemiparesis with increased muscle tone. Bilateral clinodactyly of the fifth fingers was noted. No additional focal neurological deficits were identified, and the remainder of the systemic examination was unremarkable.

The patient was born at term following an uncomplicated pregnancy and required brief neonatal intensive care admission for transient respiratory distress and a small atrial septal defect. Reduced spontaneous use of the right upper limb with early left-hand preference was first recognized at nine months of age. Persistent right-sided motor impairment prompted neurological and hematological evaluation, which demonstrated right spastic hemiparesis, MRI findings consistent with a remote ischemic insult, sickle cell trait, and a heterozygous F5 variant associated with activated protein C resistance.

Diagnostic assessment

Perinatal history revealed delivery at 40 weeks’ gestation via spontaneous vaginal delivery, with Apgar scores of 8, 9, and 9 at one, five, and 10 minutes, respectively. Birth weight was 3.3 kg, and head circumference was 34 cm. Neonatal intensive care unit admission lasted seven days, after which the patient was discharged in stable condition.

**Figure 1:**
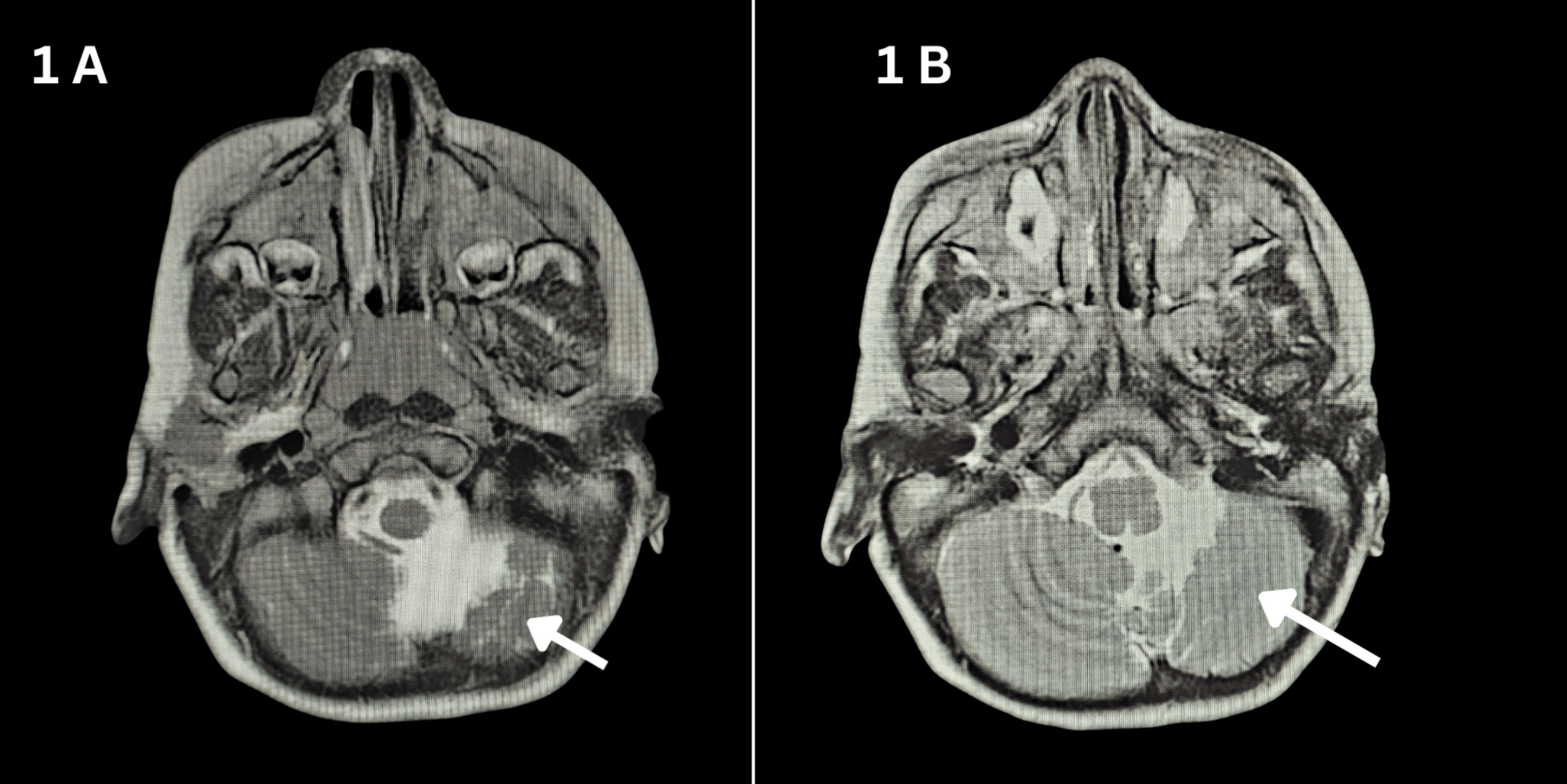
Axial T2-weighted brain MRI showing left cerebellar hemiatrophy. (A, B) Selected posterior fossa axial T2-weighted images demonstrate relative volume loss of the left cerebellar hemisphere compared with the right side, consistent with chronic left cerebellar hemiatrophy. The imaging findings support a remote ischemic insult and correlate clinically with the patient’s right-sided spastic hemiparesis.

Magnetic resonance imaging of the brain demonstrated left cerebral and cerebellar volume loss, consistent with a remote ischemic insult. Selected axial T2-weighted posterior fossa images demonstrated left cerebellar hemiatrophy (Figure 1).

Hematological evaluation confirmed sickle cell trait, without features of sickle cell disease. Whole exome sequencing identified a heterozygous c.3482G>A (p.Arg1161Gln) variant in the F5 gene (OMIM: 612309), classified as a variant of uncertain significance and associated with activated protein C resistance.

Based on clinical presentation, neuroimaging findings, and genetic evaluation, the patient was diagnosed with right-sided spastic hemiparesis secondary to probable perinatal or early-life ischemic brain injury, in association with a heterozygous F5 gene variant and sickle cell trait. Mild dysmorphic features and speech delay were also noted.

Therapeutic intervention

In the absence of documented thrombotic events and given the heterozygous nature of the F5 variant, long-term anticoagulation therapy was not initiated. Supportive management and rehabilitative care were provided.

Follow-up and outcomes

The patient remains under regular follow-up with the pediatric hematology service. The family received counseling regarding thrombosis risk and the need for prophylactic anticoagulation during high-risk situations, such as major surgical procedures or severe intercurrent illness. Gradual functional improvement has been observed over time with continued supportive care.

## Discussion

Perinatal arterial ischemic stroke (PAIS) and early-life cerebrovascular injury are well-established causes of long-term neurological sequelae in children, most commonly manifesting as unilateral spastic hemiparesis, developmental delay, and epilepsy. Unlike adult stroke, pediatric cerebrovascular disease is frequently multifactorial, and in many cases, a single unifying etiology cannot be identified. Current guidelines emphasize that delayed recognition is common, as neonatal symptoms may be subtle or transient, with the neurological phenotype becoming apparent only with later motor development [1,2].

In the present case, early hand preference noted at nine months of age, persistent right-sided spastic hemiparesis, and MRI findings of unilateral cerebral and cerebellar hemispheric volume loss with focal thalamic involvement are highly consistent with a remote perinatal or early-infantile ischemic insult. Such neuroimaging patterns are characteristic of PAIS survivors and reflect chronic sequelae rather than acute pathology [1,2,5]. The absence of documented acute neonatal stroke symptoms does not exclude this diagnosis and aligns with existing literature describing delayed clinical recognition in a substantial proportion of cases [5].

Inherited thrombophilia has been increasingly investigated as a potential contributing factor in pediatric arterial ischemic stroke, particularly in children without clear perinatal hypoxic, infectious, or traumatic triggers. A systematic review and meta-analysis demonstrated that inherited prothrombotic traits are associated with an increased risk of arterial ischemic stroke and cerebral sinovenous thrombosis in neonates and children, supporting a contributory, rather than deterministic, role for thrombophilia in pediatric cerebrovascular disease [6]. Importantly, this association appears strongest when thrombophilia coexists with additional risk modifiers.

The heterozygous F5 c.3482G>A (p.Arg1161Gln) variant identified in this patient involves a gene central to coagulation regulation through the activated protein C pathway. Variants affecting F5 function have been associated with impaired anticoagulant activity and enhanced thrombin generation, mechanisms that plausibly increase susceptibility to thrombotic events during periods of physiological stress [3,7]. Although this variant was classified as a variant of uncertain significance, its biological context supports cautious interpretation as a potential risk modifier rather than a definitive causal factor. Pediatric stroke often follows a “multiple-hit” model where genetic factors such as the F5 variant and sickle cell trait may converge with perinatal stressors, such as neonatal respiratory distress, to reach a critical thrombotic threshold [1]. Pediatric studies have reported an overrepresentation of F5-related thrombophilic traits among children with ischemic stroke compared with controls, although penetrance and clinical expression remain variable [3,6,7].

However, the clinical interpretation of thrombophilia in perinatal stroke remains controversial. Population-based prospective studies have shown that the prevalence of inherited thrombophilia among children with remote perinatal stroke phenotypes may not differ significantly from the general population, raising questions regarding causality and the utility of routine thrombophilia screening in all cases [8]. This underscores the importance of contextualizing genetic findings within the overall clinical, radiological, and developmental framework rather than attributing causation based on genotype alone.

Sickle cell trait is traditionally regarded as a benign carrier state; nevertheless, robust epidemiological evidence demonstrates an association with increased risk of venous thromboembolism [4]. While data linking sickle cell trait to arterial ischemic stroke are less consistent, biologically plausible mechanisms, including increased blood viscosity, endothelial activation, and hypercoagulability under stress conditions, support its role as a potential vascular risk modifier [9]. In the present case, the coexistence of sickle cell trait and an F5-associated prothrombotic tendency may represent a cumulative-risk constellation that increases vulnerability to early-life cerebral ischemia, even in the absence of recurrent thrombotic events.

The patient’s stable clinical course and absence of recurrent thrombosis support the conservative management approach adopted. Current pediatric stroke and thrombosis guidelines advise against routine long-term anticoagulation in asymptomatic carriers of inherited thrombophilia, recommending instead individualized risk stratification and prophylaxis during high-risk situations such as major surgery, prolonged immobilization, or severe intercurrent illness [1,10]. Long-term outcome in pediatric arterial ischemic stroke is primarily determined by the extent and location of the initial brain injury rather than ongoing thrombotic activity [5,10].

Overall, this case reinforces the multifactorial nature of pediatric cerebrovascular disease and highlights the importance of careful interpretation of genetic findings, particularly variants of uncertain significance. It illustrates the value of comprehensive etiological evaluation, balanced clinical judgment, and long-term multidisciplinary follow-up. Further research integrating genetic data with neuroimaging and longitudinal outcomes is required to refine genotype-phenotype correlations and guide evidence-based risk stratification in pediatric stroke [11,12].

## Conclusions

This case highlights the possible contribution of the c.3482G>A F5 variant, classified as a variant of uncertain significance, as a potential risk modifier in early-life ischemic stroke. The coexistence of this F5 variant with sickle cell trait may have lowered the thrombotic threshold in this patient, supporting the value of comprehensive etiological and genetic evaluation in selected children with otherwise unexplained cerebrovascular injury.

Management of such patients should be individualized, with careful interpretation of genetic findings, especially variants of uncertain significance, within the broader clinical context. Long-term multidisciplinary follow-up and situational, risk-based thromboprophylaxis during high-risk periods remain central to optimizing neurological outcomes while avoiding unnecessary long-term anticoagulation.
